# Immunologic response to first booster vaccination in dogs treated with zenrelia™ (ilunocitinib tablets) at up to three times the recommended therapeutic dose compared to untreated controls

**DOI:** 10.1186/s12917-025-04929-z

**Published:** 2025-07-22

**Authors:** Genevieve M. Fent, Jay Jacela, Rodrigo Plazola-Ortiz, Jeremiah Olps, Erin E. McCandless, Céline E. Toutain, Sandra O’Kelley, Stephen King

**Affiliations:** 1 Elanco Animal Health, Greenfield, IN USA; 2 Erin McCandless Consulting LLC, Dexter, MI USA; 3 Elanco Animal Health, Crisco Uno, Bâtiment C, 3-5 avenue de la Cristallerie, Sèvres, Cedex, 80022 -92317 CS France

**Keywords:** JAK inhibitor, Booster, Vaccination, Ilunocitinib, Zenrelia™, Antibody response

## Abstract

**Background:**

This prospective, non-inferiority study was designed to evaluate the serologic response to booster vaccination in dogs previously vaccinated according to standard of care practice and treated for 56 days with up to 3X the recommended label dose of ilunocitinib. Vaccination occurred 28 days post-treatment initiation and continued through day 56. Measured study parameters included: vaccine-induced serum antibody titers at 15- and 28-day post-vaccination, along with hematological and clinical chemistry parameters.

**Results:**

Serum antibody titer levels were not significantly different between the control and ilunocitinib treated groups (1X and 3X), and both met the non-inferiority criteria versus the control group after 56 days of treatment (SD 56). All dogs administered ilunocitinib had titers at or above threshold for rabies, CAV-2, and CPV at 15- and 28-day post-vaccination timepoints (SD 43 and SD 56, respectively). The percentage of dogs with titers at or above threshold on SD 43 for CDV were 96, 100, and 95% in the control, 1X and 3X groups, respectively. By SD 56, the percentage of animals with titers above threshold for CDV were similar to pre-vaccination levels in all groups.

**Conclusion:**

This study demonstrated treatment with ilunocitinib at 1X or 3X the therapeutic dose for 56 days did not significantly attenuate the serologic response to CAV-2, CPV, CDV or rabies booster vaccinations compared to untreated control animals. All animals remained clinically healthy during the study, with only mild gastrointestinal or skin abnormalities typical of laboratory dogs or JAK inhibitor therapy.

## Background

Atopic Dermatitis (AD) is a complex clinical syndrome affecting up to 15% of the canine population, often requiring lifelong treatment [1]. Janus Kinase (JAK) inhibitors have been safely and efficaciously used to treat multiple inflammatory diseases in humans [2] and dogs [3], specifically AD in the latter. The JAK family of proteins are expressed in a wide variety of immune cells and are critical mediators of cytokine signaling [4]. JAK inhibitors mitigate dysregulated inflammation in the disease state, influencing immune system responses.

Ilunocitinib is a JAK inhibitor shown to rapidly and safely control canine AD. In a field efficacy and safety study of ilunocitinib, 94–97% of dogs had a 50% reduction in baseline Canine Atopic Dermatitis Extent and Severity Index-4 (CADESI-4) scores between days 28–112 of treatment and 69% of dogs had achieved clinical remission (i.e. CADESI-4 score < 10) by Day 112 [5]. AD in dogs often requires long-term therapy so it is important to understand how therapeutics with immune modulating mechanisms, such as ilunocitinib, may affect vaccine response.

Vaccinations play a critical role in the health and wellness of dogs by providing protection from infectious diseases capable of causing illness and death [6]. A core program including canine distemper virus (CDV), canine parvovirus (CPV) and canine adenovirus type − 2 (CAV-2) is recommended for all dogs worldwide [7]. Rabies and leptospirosis may also be considered part of the core vaccination program depending on region of the world in which the animal resides [7]. Vaccination of pet dogs is a crucial element in supporting a One Health philosophy by limiting the reservoir for zoonotic pathogens like rabies that are fatal to humans [8]. Although the immunological response to vaccination encompasses highly complex and diverse mechanisms, titer measurement is a commonly used biomarker to assess the antibody component of the vaccine response.

The safety of ilunocitinib has also been evaluated in multiple laboratory studies in dogs [5, 9], including a 6-month laboratory study at daily doses of 1, 2, 3, and 5X the maximum recommended dose compared to sham-dosed control dogs [9]. This study demonstrated a favorable safety profile with no clinically significant changes at the therapeutic dose. Effects at elevated doses were minimal and consistent with known effects of JAK inhibition, including changes in hematological parameters, total protein, and fibrinogen. Immune cells were monitored throughout the study and no clinically relevant changes in leucocyte cell population were detected based on hematology and immunophenotyping data [9].

Vaccine response was evaluated in a separate study where 10-month-old, vaccine naïve, dogs were treated with 3X the maximum therapeutic dose, immunized with the core panel of modified live and killed vaccines and compared to untreated controls. Although the intent of the study was to use healthy dogs, the study was confounded by pathogen exposure and infectious disease outbreaks, therefore impacting the study’s interpretability. Under the actual study conditions, where infectious disease was confirmed, treated dogs had increased morbidity and mortality which may not be unexpected due to the mechanism of action of JAK inhibition. Despite this, vaccines were still administered and all surviving dogs in both treatment groups achieved the target titer level for core antigens after primary modified live vaccination on day 28 [10]. Rabies titers met threshold levels in 5/6 treated dogs but not until day 116 when the animals had recovered from infectious disease [10]. Based on the limited interpretability of the confounded vaccine study, combined with the criticality of understanding how treatment with ilunocitinib affects antibody response to vaccination, the current study was conducted to assess ilunocitinib’s impact on antibody response to clinically relevant booster vaccinations.

This study evaluated vaccine booster responses which are most relevant to the intended patient population for ilunocitinib, adult dogs, who have been previously vaccinated according to standard of care practice. The study endpoints were vaccine-induced antibody titers, immunophenotyping, and clinical assessments.

## Methods

### Objectives and standards

The objective of this study was to evaluate the serologic response to core booster vaccination in dogs previously vaccinated with CDV, CPV, CAV-2, and rabies and assigned to one of three treatment groups: untreated controls or treated with 1X or 3X the recommended label dose of ilunocitinib.

The animal phase of this study was conducted in accordance with Good Research Practice (GRP). The animal care and use were reviewed, and the conduct of the study approved by the Elanco Institutional Animal Care and Use Committee prior to study initiation. All protocol amendments impacting animal welfare were also approved by the Institutional Animal Care and Use Committee. The study was executed in compliance with the US Code of Federal Regulations Title 9, Parts 1–4, The Animal Welfare Regulations; US Code of Federal Regulations Title 7, Chap. 54, Sects. 2131–2159, The Animal Welfare Act and followed the Guide for the Care and Use of Laboratory Animals.

### Animals and maintenance

A total of 72 (33 males and 39 females) 10–11-month-old Beagle dogs fully vaccinated as puppies against CDV, CPV, CAV-2, and rabies, and due for their first booster (6-months since last vaccination), were purchased from a commercial laboratory animal supplier. Animals were acclimatized at the study site for 28 days prior to study initiation.

Dogs were pair-housed in three separate airspaces (except for 2 dogs following removal of pen mate prior to study day 0) with up to 24 dogs per airspace. Each pen had approximately 48 square feet. Upon arrival, dogs were placed in their assigned pens in pairs based on study randomization by treatment group (same treatment group per pen) and sex (same sex per pen, except one pair that consisted of one male and one female dog) within pens in a single indoor Animal Biosafety Level 2 (ABSL2) facility. Each pair was housed together in 2 adjacent pens with the gate between pens open to allow the pair free access and movement between pens. At a minimum, pens were clearly labeled with pen ID and blinded animal number.

From SD 0 to 55, dogs were separated each day for a short period of time just before and after dosing. All dogs were individually identified by a unique ear tattoo and microchip. Dogs were housed in a controlled environment that provided temperature, ventilation, and light cycle appropriate for their age. Dogs had *ad libitum* access to potable water and a laboratory canine diet. All dogs were provided with environmental enrichment and socialization.

### Allocation to groups

This was a randomized, masked, negative-controlled non-inferiority study with a three-arm parallel design. Prior to arrival, a total of 72 dogs initially identified for inclusion in the study were randomized, by sex (39 females and 33 males), into either the control group (Group 1), 1X group (Group 2, 0.6–0.79 mg/kg/day ilunocitinib), or 3X (Group 3, 1.81–2.26 mg/kg/day ilunocitinib) taking into consideration pair housing. Animals were assigned to pairs, and the paired animals were randomized to treatment groups, following a generalized randomized block design, stratified by sex and pre-study pair average antigen titer levels. Rooms were used as a blocking factor with each block (room) having 24 animals. Pairs were then assigned to pens within a room following a completely randomized design performed by the Statistician using SAS^®^ software version 9.4. Masked and unmasked randomization lists were created and distributed only to the appropriate personnel; the unmasked randomization plan did not include treatment group assignment information and was shared before the arrival of animals to the site. Prior to treatment initiation on SD 0, four dogs (one from the control and three from the ilunocitinib 3X group) were withdrawn due to chronic health issues; as a result, 23 dogs in the control group, 24 dogs in the 1X group, and 21 dogs in the 3X group remained on study.

### Investigational veterinary product administration or Sham dosing

Animals were separated into individual cages by closing the gate between the pair housing cages prior to dosing. Dogs in the 1X and 3X groups were orally administered the appropriate number of ilunocitinib tablets, using spray cheese (Easy Cheese^®^, Mondelez International East Hanover, NJ, USA), based on SD -4 body weight (BW). Doses were manually administered, and the inside of the mouth was immediately inspected to ensure tablets had been swallowed. After all the dogs in the room were dosed, the gate between each paired housing was opened.

The control group was sham-dosed by hand-feeding a small amount of spray cheese (Easy Cheese^®^, Mondelez International East Hanover, NJ, USA).

### Vaccines and vaccination

Commercially purchased vaccines, Nobivac^®^ Canine 1-DAPPV (Merck Animal Health, Rahway, NJ, USA) and IMRAB^®^ 3 (Boehringer Ingelheim Animal Health, Athens, GA, USA), were administered as per label directions provided on the package inserts on SD 28. Vaccines were administered subcutaneously in the shoulder area (Nobivac Canine 1-DAPPV on the left and IMRAB 3 on the right) at a dose of 1.0 mL per dog.

### Assessments and variables recorded

Animals were observed at least once daily throughout the study. Observations included general behavior, body systems, feces (color, consistency, presence of blood and mucus), locomotion, and other symptoms such as abnormal urine, salivation, vomiting, and presence of blood. Body weights were recorded on SD -4.

Blood was collected for clinical chemistry evaluation on SD -1, 28 and 56. Blood was collected for complete blood count (CBC) and immunophenotyping analysis on study days − 1, 28, 43 and 56. For pharmacokinetic analysis, blood was collected on study days 6 and 50.

### Serum titers against vaccine antigens

Antibody titers against CDV and CAV-2 were measured using serum neutralization assays, and titers against CPV were measured using a hemagglutination inhibition assay. Rabies antibody titers were determined using the Rapid Fluorescent Focus Inhibition Test (RFFIT). The study design is shown in Table 1.

**Table 1:** Study design

Treatment Group	Treatment	Number of Animals	Dose (mg/kg)
1	Control	23	0
2	Ilunocitinib 1X (IVP)	24	0.60–0.79
3	Ilunocitinib 3X (IVP)	21	1.81–2.26

Whole blood was collected from all animals into a 6-mL serum separator tube and allowed to sit at room temperature for at least one hour prior to centrifugation for 20 min at 1300 × g at 20 °C. The serum was then separated into 3 aliquots and frozen. Serum was tested at the study site laboratory for CAV-2, CDV, and CPV (SD -1 only) titers. Subsequent CPV hemagglutination inhibition (HI) testing was conducted at the Animal Health Diagnostic Center of Cornell University, Ithaca, NY, USA. Rabies titers were performed by Atlanta Health Associates, Inc, Cumming, GA, USA.

### Statistical analysis of the data

All statistical analyses were conducted using SAS software version 9.4. All animal data were summarized through descriptive statistics or frequency counts. All assessments were evaluated as a two-tailed test at the 0.05 level of significance unless otherwise stated. The experimental unit was the pen.

### Statistical power

Power estimations were performed to determine the sample size needed to demonstrate non-inferiority for three co-primary endpoints. These endpoints corresponded to three antigen titer variables (CAV-2, CDV, CPV). A one-sided alpha of 0.025 was used for each comparison. For each variable, a simulation study with 1000 replicates was conducted using SAS software version 9.4. Distribution parameters (mean and standard deviation) for the simulations were derived from data obtained in a previous study. Simulations for CAV-2, CDV, and CPV titer were based on changes from baseline. The cage, with two animals per cage, was considered the experimental unit for the simulation study.

Multiple scenarios were simulated to evaluate the statistical power with different numbers of cages. The simulation study demonstrated that 12 cages with two dogs in each (24 dogs total per room and 72 dogs overall) by treatment group were needed to achieve a power above 90% to demonstrate non-inferiority, using a non-inferiority margin of 2 on the log2 scale between treatment groups for all three variables. A non-inferiority margin of 2 on log2 scale was chosen as a clinically meaningful difference based on assay variability of plus or minus one assay dilution. Rabies was excluded from these calculations due to high assay variability.

### Non-inferiority analysis - serum antibody titers

The log-transformed antibody titers were analyzed using a Repeated Measures Analysis of Covariance model (RMANCOVA). The fixed effects in the statistical model included baseline titer (pre-vaccination Day 28 titers), treatment (TRT), time (TIME), sex (SEX), and their two-way interactions, TRT*SEX, TRT*TIME and TIME*SEX; and the three-way interaction TRT*TIME*SEX. Room and Room-by-Treatment were used as random effects. The following covariance structures were considered: compound symmetry (CS), first order autoregressive (AR(1)), and unstructured (UN). The model with the lowest Akaike Information Criteria (AIC) was retained. From the selected model, the least square means (on log scale) and 95% Confidence intervals (CI) were estimated.

Non-inferiority of each treatment group compared to the control group on SD 56 was assessed based on the least squares mean (LSM) difference and the 95% CI using a margin of 2 on logscale. If the upper limit of the 95% CI for the treatment difference (Control - treated) was less than 2 then the treatment was declared non-inferior to control.

### Statical analysis of additional endpoints

For endpoints measured pre-treatment (baseline) and multiple times during the study (logarithmically transformed vaccine titers, clinical chemistry, and hematology parameters), a RMANCOVA model was used for analysis. The fixed effects in the statistical model included a baseline measurement, treatment (TRT), time (TIME) sex (SEX) and the two-way and 3-way interactions. Room and Room-by-Treatment were included as random effects. The following covariance structures were considered: compound symmetry (CS), first order autoregressive (AR(1)) and unstructured (UN). The model with the lowest AIC was retained.

If the 3-way interaction (TRT*TIME*SEX) was significant (α = 0.05), then only descriptive statistics were presented for each treatment group at each time point within each sex, and no further analyses were conducted. If the 3-way interaction was not significant, the following stepwise process was followed:

- Treatment-by-sex (TRT*SEX) and the treatment-by-time (TRT*TIME) interactions (α = 0.10) were evaluated. If the treatment-by-sex interaction was significant, treatment means were compared to control for each sex. If the treatment-by-time interaction was significant, treatment means were compared to control for each time point. If neither interaction was significant, the next step below was then followed.
- If no interaction terms were significant, TRT main effect (α = 0.10) was evaluated. If the main effect was significant, treatment means were compared to the control across the pooled sex and time points.
- If no interaction terms were significant and the TRT main effect was not significant, no further evaluations were conducted.

To compare vaccine titer results to protective titer levels reported in the literature, the number and percentage of animals with titers exceeding the protective titer levels were determined and presented by treatment group and study day.

## Results

### Animal observations

Physical examination and daily observation findings were mild and related to gastrointestinal and integumentary systems. Soft feces, diarrhea, vomiting, and otitis were seen in all groups. Three or four dogs in the 1X or 3X group, respectively, had skin lesions consistent with JAKi treatment (interdigital furunculosis or dermal nodule). All but two lesions (1 in each group) resolved with or without treatment prior to the end of the study.

### Pharmacokinetic analysis

Concentrations of ilunocitinib in the control group were below the limit of quantitation (0.5 ng/mL) in all dogs when tested on SDs 6 and 50. Mean plasma concentrations of ilunocitinib in the 1X group were 200.2 (SD = 70.7, Range = 96.8–371) and 183.2 ng/mL (SD = 71.9, Range = 58.4–334) on SDs 6 and 50, respectively. Mean plasma concentrations of ilunocitinib in the 3X group were 542.0 (SD = 164, Range = 267–808) and 475.1 ng/mL (SD = 148, Range = 199–660) on SDs 6 and 50, respectively. This analysis confirmed that control dogs were not exposed to ilunocitinib, and treated dogs were exposed to an appropriate level of ilunocitinib relative to their respective treatment groups.

### Clinical pathology

There were no treatment-related effects on serum chemistry or hematology parameters.

### Serologic response to booster vaccination

The mean serum titers on SD 56, the primary endpoint, and the results of the non-inferiority analysis are shown in Table 2. The serum titer levels were not significantly different between the control and treated groups and both treated groups (1X and 3X) met the non-inferiority criteria versus the control group.

**Table 2:** Mean serum titers on SD 56 and results of Non-Inferiority analysis using RMANCOVA

Viral Antigen	Sex	Treatment	Geometric Mean Titer (LSM)	Difference in LS means Estimate (Control -IVP)	Standard Error of the Difference	95% CI	*p*-value vs. Control	Non-Inferiority vs. Control
Rabies^1^	F	0X	20.448	-	-	-	-	-
	M	0X	15.864	-	-	-	-	-
	F	1X	16.263	0.142	0.257	(-0.374, 0.659)	0.582	YES^4^
	M	1X	17.603	-0.065	0.285	(-0.638, 0.509)	0.822	YES^4^
	F	3X	9.030	0.508	0.276	(-0.047, 1.063)	0.072	YES^4^
	M	3X	9.353	0.328	0.283	(-0.241, 0.898)	0.252	YES^4^
CAV-2^2^	P	0X	1116.339	-	-	-	-	-
	P	1X	1561.363	-0.484	0.391	(-1.271, 0.303)	0.222	YES^3^
	P	3X	1458.170	-0.385	0.410	(-1.209, 0.438)	0.351	YES^3^
CDV^2^	F	0X	100.380	-	-	-	-	-
	M	0X	80.963	-	-	-	-	-
	F	1X	99.282	0.016	0.420	(-0.828, 0.860)	0.970	YES^4^
	M	1X	78.183	0.050	0.485	(-0.923, 1.024)	0.918	YES^4^
	F	3X	75.921	0.403	0.473	(-0.548, 1.354)	0.399	YES^4^
	M	3X	60.988	0.409	0.484	(-0.563, 1.380)	0.402	YES^4^
CPV^2^	P	0X	595.567	-	-	-	-	-
	P	1X	662.004	-0.153	0.327	(-0.809, 0.504)	0.642	YES^3^
	P	3X	613.776	-0.043	0.340	(-0.726, 0.639)	0.899	YES^3^

^1^Values presented on log base 5 scale, ^2^Values presented on a log base 2 scale, ^3^non-inferiority vs. Control assessed for pooled sex

^4^Non-inferiority vs. Control assessed for each sex, Estimates derived using RMANCOVA: Baseline (Day 28) titers are included as a covariate, F = female, M = male, P = pooled

The mean serum titers on SD 43 are presented in Table 3 and were not significantly different between the control and treated groups, except for CPV titers which were higher in the 1X treated group compared to control group.

**Table 3:** Mean serum titers on SD 43 and results of RMANCOVA analysis

Viral Antigen	Sex	Treatment	Geometric Mean Titer (LSM)	Difference in LS means Estimate (Control - IVP)	Standard Error of the Difference	95% CI	*p*-value vs. Control
**Rabies** ^**1**^	F	0X	35.674	-	-	-	-
	M	0X	53.569	-	-	-	-
	F	1X	38.141	-0.042	0.257	(-0.558, 0.475)	0.872
	M	1X	33.379	0.294	0.285	(-0.279, 0.867)	0.308
	F	3X	24.405	0.236	0.276	(-0.319, 0.791)	0.397
	M	3X	25.390	0.464	0.283	(-0.106, 1.033)	0.108
**CAV-2** ^**2**^	P	0X	1360.827	-	-	-	-
	P	1X	1478.571	-0.120	0.391	(-0.906, 0.667)	0.761
	P	3X	1408.467	-0.050	0.410	(-0.873, 0.774)	0.904
**CDV** ^**2**^	F	0X	173.052	-	-	-	
	M	0X	180.271	-	-	-	-
	F	1X	193.797	-0.163	0.355	(-0.878, 0.551)	0.648
	M	1X	173.694	0.054	0.406	(-0.762, 0.869)	0.895
	F	3X	206.740	-0.257	0.405	(-1.070, 0.557)	0.529
	M	3X	109.378	0.721	0.405	(-0.093, 1.534)	0.081
**CPV** ^**2**^	P	0X	762.854	-	-	-	-
	P	1X	1217.129	-0.674	0.327	(-1.330, -0.018)	0.044
	P	3X	997.082	-0.386	0.340	(-1.069, 0.296)	0.261

^1^Values presented on log base 5 scale, ^2^Values presented on a log base 2 scale, Estimates derived using RMANCOVA: Baseline (Day 28) titers are included as a covariate, F = female, M = male, P = pooled

The titer response in individual dogs was also compared to protective titer levels reported in the literature for the individual antigens tested [11, 12]. These protective titer levels (henceforth referred to as thresholds) are shown in Table 4. The frequency of dogs with a titer at or above threshold by treatment group and SD is presented in Table 5. All dogs in the ilunocitinib 1X and 3X dose groups had titers at or above threshold for rabies, CAV-2, and CPV on SD 43 and SD 56. On SD 43, CDV titers in 96, 100, and 95% of dogs in control, 1X and 3X groups, respectively, were at or above threshold. By SD 56, the percentage of animals with titers above threshold for CDV were similar to pre-vaccination (SD 28) levels in all groups.

**Table 4:** Titer thresholds

Virus	Analysis performed to determine titer level	Threshold for protective immunity
Canine Distemper Virus (CDV)	Serum Neutralization	≥ 32
Canine Parvovirus (CPV)	Hemagglutination Inhibition	≥ 80
Canine Adenovirus (CAV-2)	Serum Neutralization	≥ 16
Rabies	Rapid Fluorescent Focus Inhibition Test (RFFIT)	> 0.5 IU/mL

**Table 5:** Frequency of dogs with titer equal to or above threshold

Viral Antigen	Treatment	Day	*N*	Number at or above Threshold	Percent *N* above Threshold
Rabies	0X	28	23	18	78
		43	23	23	100
		56	23	23	100
	1X	28	24	12	50
		43	24	24	100
		56	24	24	100
	3X	28	21	11	52
		43	21	21	100
		56	21	21	100
CAV-2	0X	28	23	22	96
		43	23	23	100
		56	23	23	100
	1X	28	24	24	100
		43	24	24	100
		56	24	24	100
	3X	28	21	19	90
		43	21	21	100
		56	21	21	100
CDV	0X	28	23	21	91
		43	23	22	96
		56	23	21	91
	1X	28	24	18	75
		43	24	24	100
		56	24	21	88
	3X	28	21	15	71
		43	21	20	95
		56	21	16	76
CPV	0X	28	23	22	96
		43	23	23	100
		56	23	23	100
	1X	28	24	23	96
		43	24	24	100
		56	24	24	100
	3X	28	21	20	95
		43	21	21	100
		56	21	21	100

## Discussion

The titer data in this study demonstrated that the serological response to vaccination for rabies, CAV-2, CDV, and CPV met the non-inferiority criteria versus the control group. This was true for both groups administered ilunocitinib at the 1X or 3X dose as assessed on SD 56. Individual animal antibody titers can be highly variable. To account for this, a non-inferiority design was used in the current study. A repeated measures analysis was performed, with baseline titers included as a covariate to account for baseline variability. The non-inferiority analysis uses 95% Confidence Intervals to determine whether the treated animals’ response is not significantly less than that of the controls by a margin of 2. The margin was selected to ensure that the results are clinically meaningful. The margin of 2 used in this study takes into account variability inherent within the titer assays themselves and ensures confidence in the interpretability of the primary study endpoint. Therefore, ilunocitinib does not significantly impact booster response to the canine core vaccines at the predetermined titer threshold levels.

In addition to variability amongst titers between individuals, there is also variability associated with the kinetics of the peak antibody titer post-vaccination. In this study, dual time points were included to ensure this diversity within the response is fully accounted for. Interestingly, for CDV, CPV and rabies the secondary timepoint of SD 43 (15 days post-vaccination) captured the highest measured antibody response as compared to the primary endpoint of SD 56 (28 days post-vaccination). This is in agreement with other canine rabies vaccine response studies and confirms the previously reported importance of capturing the appropriate kinetic window when assessing antibody titers [13]. This study was designed to capture peak titer results so additional studies would be required to determine if therapeutic JAK inhibition impacted long-term kinetics of titer levels.

Associated with the highest titer response measured at SD 43 is a trend towards a reduced titer at SD 56. While this decrease is not statistically significant, it is most noticeable for the rabies vaccine. The World Health Organization, who established the protective titer level threshold for rabies vaccination, recommends re-screening of titers every 6 months in situations where there is permanent risk of rabies exposure. This highlights the expectation for dynamically decreasing levels of titers within a short time period. Studies examining antibody retention support that expectation, demonstrating rabies titers in dogs decline over time [14, 15]. Additionally, antibody titers are not the only immunological component conferring protection as cell mediated responses contribute to vaccine-induced rabies protection as well [16]. Taken together, antibody titers should be viewed as a transient biomarker of vaccination as opposed to a measure of the totality of immune protection [17].

Understanding impacts on the immune system is a critical aspect of the evaluation of any immune-modulating therapeutic and a key component of the assessment of the safety of new drugs. The current study utilized a statistically robust, negative-controlled non-inferiority design to assess the impact of ilunocitinib on the immune system’s ability to produce an antibody recall response to the canine core vaccine panel. Booster responses were evaluated to best represent the anticipated canine AD patient population who would most likely not be naïve to vaccines. Elevated doses of drug and inclusion of additional clinical endpoints were employed to ensure that any drug-related effects would be visible. Pharmacokinetic assessment confirmed treated groups received the intended dose. The results demonstrate that in both 1X and 3X dose groups ilunocitinib does not significantly attenuate the serological response against canine core and killed rabies booster vaccinations in dogs. Additionally, no biologically relevant changes in secondary measures of hematological or serum chemistry profiles occurred.

## Conclusion

This study demonstrates treatment with ilunocitinib at 1X or 3X the therapeutic dose for 56 days does not significantly attenuate the response to CAV-2, CPV, CDV or rabies booster vaccinations compared to untreated control animals. This study adds to the growing body of literature supporting the safe use of ilunocitinib, the newest member of the JAK inhibitor class available to veterinarians to treat canine AD.

## Data Availability

The data supporting the conclusions of this article are included within the article.

## Abbreviations

AD: Allergic and atopic dermatitis
JAK: Janus-Kinase
CADESI-4: Canine Atopic Dermatitis Extent and Severity Index-4
3X: Three times
CDV: Canine distemper virus
CPV: Canine parvovirus
CAV-2: Canine adenovirus-2
GRP: Good Research Practice
SD: Study day
ABSL2: Animal Biosafety Level 2
BW: Body weight
CBC: Complete blood count
RFFIT: Rapid Fluorescent Focus Inhibition Test
HI: Hemagglutination inhibition
RMANCOVA: Repeated Measures Analysis of Covariance model
TRT: Treatment
CS: Compound symmetry
AR: Autoregressive
UN: Unstructured
AIC: Akaike Information Criteria
CI: Confidence intervals
LSM: Least squares mean
GMT: Geometric Mean Titer
1X: One times
3X: Three times
SD: Study day
CS: Compound symmetry
